# Unilateral biportal endoscopic lumbar interbody fusion vs. posterior lumbar interbody fusion for the treatment of bilateral lumbar spinal stenosis

**DOI:** 10.3389/fsurg.2025.1533458

**Published:** 2025-06-30

**Authors:** Renjie Dong, Shan Wu, Dian Zhong, Guosheng Zhao, Yang Liu, Yang Wang

**Affiliations:** Department of Spine Surgery, The Second Affiliated Hospital of Chongqing Medical University, Chongqing, China

**Keywords:** unilateral biportal endoscopic lumbar interbody fusion, bilateral decompression, posterior lumbar interbody fusion, lumbar spinal stenosis, lumbar spine instability

## Abstract

**Objective:**

This study aimed to evaluate the clinical efficacy and safety of unilateral biportal endoscopic lumbar interbody fusion (UBE-LIF) and posterior lumbar interbody fusion (PLIF) for treating patients with lumbar spinal stenosis with bilateral radiating symptoms. All the patients included in the study had single-segment lumbar spinal stenosis.

**Methods:**

From January 2021 to June 2023, 21 patients with lumbar spinal stenosis treated with UBE-LIF and 29 patients with lumbar spinal stenosis treated with PLIF were retrospectively analyzed. Clinical differences in demographic characteristics, surgical details, preoperative and postoperative visual analog scale (VAS) scores, Oswestry Disability Index (ODI) score, MacNab criteria, and complications were analyzed.

**Results:**

A total of 50 patients were included in this study. There were no differences in demographic characteristics and MacNab criteria between the two groups (*P* > 0.05). The operation time in the UBE-LIF group was significantly longer than that in the PLIF group (*P* < 0.05). Hemoglobin loss in the UBE-LIF group was significantly lower than the PLIF group, as were postoperative drainage volume and hospitalization days (*P* < 0.05). The postoperative VAS and ODI scores were significantly lower than those before the respective operations in both groups. The VAS and ODI scores in the UBE-LIF group were significantly lower than those in the PLIF group 1 week after the respective operations (*P* < 0.05).

**Conclusions:**

UBE-LIF was able to achieve the same bilateral decompression as PLIF and resulted in better symptomatic improvement in the early postoperative period, which may be related to it resulting in less damage to the back muscle tissue. This study suggests that UBE-LIF may be a minimally invasive alternative to PLIF.

## Introduction

Lumbar spinal stenosis (LSS) is a clinical syndrome of buttock or lower limb pain that may be accompanied by lower back pain (1). It is usually due to the narrowing of the central spinal canal and/or intervertebral foramen, resulting in compression of the corresponding nerve roots and other adjacent structures. Degenerative changes in the lumbar spine are a major etiological factor in LSS. Generally, degenerative LSS is associated with facet joint hypertrophy, disc bulging, osteophyte formation, and hypertrophy of ligamentum flavum. Accordingly, in addition to pain, patients with LSS experience lower back pain, unilateral or bilateral lower extremity numbness, and neurogenic claudication (2). For the majority of people, non-surgical interventions are first considered and treat symptoms well. Once regular and adequate conservative treatment is ineffective, surgical treatment is a reasonable alternative. Approximately 60%–85% of patients have a satisfactory clinical outcome after surgical treatment (3). With advancements in new technologies, the indications for lumbar fusion continue to expand. The minimally invasive technique of fusion surgery also reduces the threshold for fusion surgery in patients with poor health and provides more options for patients who are considered too weak to undergo traditional open surgery (4).

The main purpose of the operation is to decompress the nerve roots, provide stability to the spine, mitigate symptoms, and improve the function of the patients. Posterior lumbar interbody fusion (PLIF) has been regarded as the gold standard in recent decades. However, it has shortcomings such as the potential for retraction injury to the thecal sac and nerve roots, insufficient treatment of lordosis, and iatrogenic injury to the paraspinal musculature and posterior ligament complex (5). Reducing approach-related complications is an issue that surgeons must consider.

With the advancements in surgical instruments and endoscopic techniques, minimally invasive surgery has become popular with its advantages of smaller wounds, less damage to paravertebral muscles, and maintenance of the integrity of the posterior column (6). In recent years, the biportal endoscopic system and unilateral biportal endoscopic lumbar interbody fusion (UBE-LIF), a new minimally invasive procedure, combine the advantages of conventional and endoscopic surgery. It provides a wide field of view for the operation as the operating elements are located in different channels and do not interfere with each other. The unilateral biportal endoscopic technique has the advantages of a larger instrument viewing angle, more flexible operation, and less damage (7–9). In contrast to the uniportal endoscopic system, the UBE-LIF working portal is used only as a portal for spinal instrumentation, thus allowing the use of various spinal instruments and endoscopic equipment (10). UBE-LIF has been effectively applied in the treatment of various lumbar diseases, including lumbar disc herniation, spinal canal or foramen stenosis decompression, and lumbar spondylolisthesis, with remarkable clinical results (11). Unilateral endoscopy laminectomy for bilateral decompression (ULBD) is usually used in the treatment of patients with bilateral nerve root symptoms and can result in the decompression of the central spinal canal and bilateral lateral recess by moving the root of the spinous process to the opposite side (12). UBE-LIF can also reduce the clinical symptoms of lower limb pain in patients with lumbar spinal stenosis. Currently, few studies compare UBE-LIF and PLIF for bilateral decompression. Therefore, the purpose of this paper is to research the clinical effect of UBE-LIF in unilateral and bilateral decompression and compare the results with PLIF.

## Methods

### Patient selection

We retrospectively reviewed the clinical data of patients with lumbar spinal stenosis who underwent UBE-LIF or PLIF surgery at the Second Affiliated Hospital of Chongqing Medical University from January 2021 to June 2023. In this study, we collected demographic information, perioperative indicators, and radiological results. The study was conducted under a protocol approved by our Institutional Review Board and informed consent was obtained for experimentation with human subjects.

The inclusion criteria were as follows: (1) the patient was diagnosed with LSS and single-segment spinal stenosis based on radiological data; (2) the patient had radicular pain and bilateral lower limb symptoms; (3) preoperative conservative treatment was ineffective for at least 6 weeks; (4) the follow-up time was at least 1 year.

**Figure 1:**
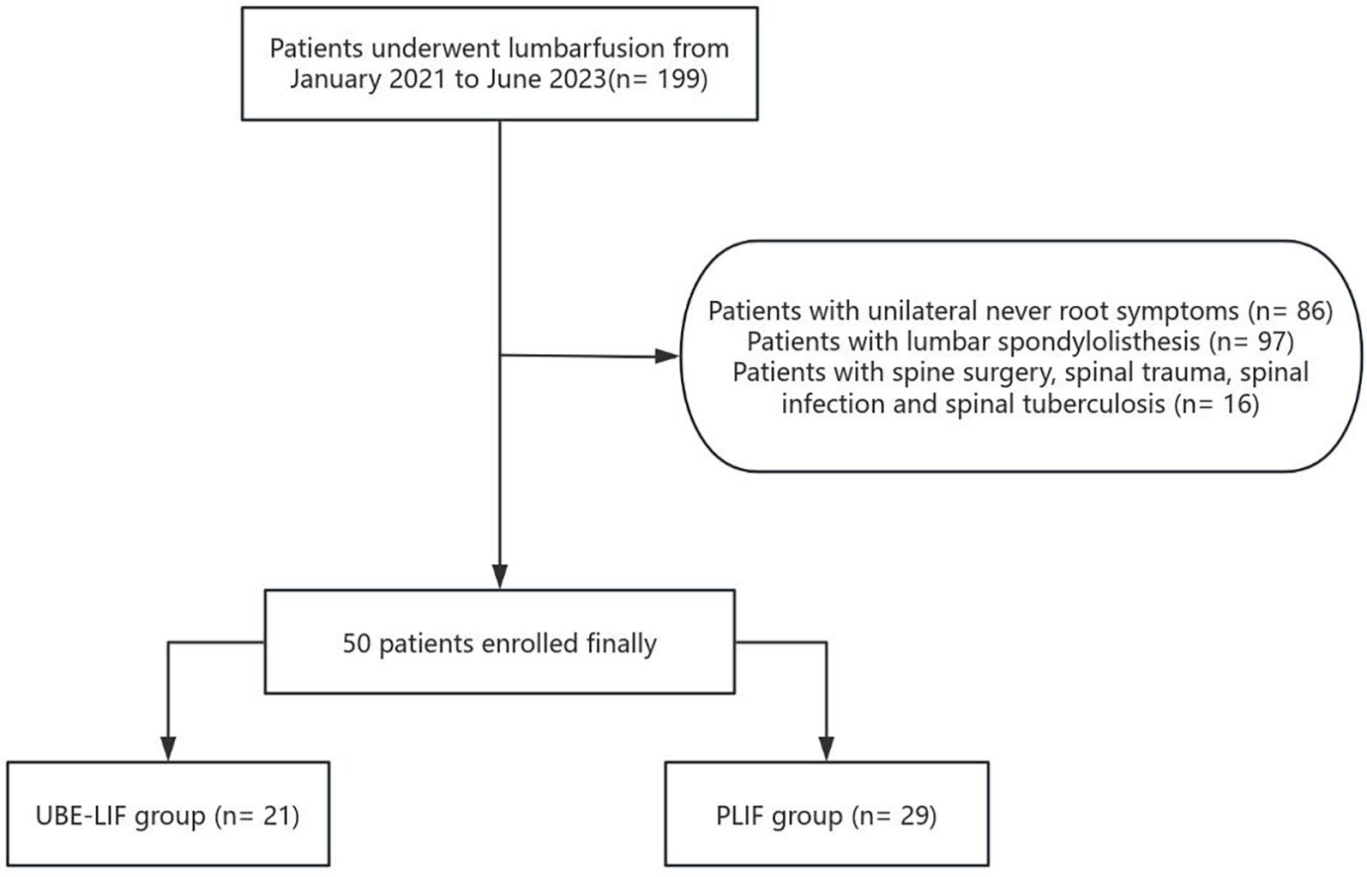
A flowchart of the patient screening process. A total of 199 patients underwent fusion surgery from January 2021 to June 2023. After the exclusion criteria were applied, 50 patients were included in the final study and divided into the UBE-LIF group and the PLIF group.

The exclusion criteria were as follows: (1) previous history of lumbar surgery; (2) combined with spinal tuberculosis, infection, or tumor; (3) nerve root symptoms in multiple segments; (4) combined lumbar spondylolisthesis; (5) incomplete or missing clinical data or poor compliance with regulations (Figure 1). Finally, 50 patients who met the criteria were included. There were 21 patients who received UBE-LIF treatment and 29 patients who received PLIF treatment.

### Surgical procedures

The two groups of patients underwent lumbar fusion, spinal canal decompression, and pedicle screw fixation.

**Figure 2:**
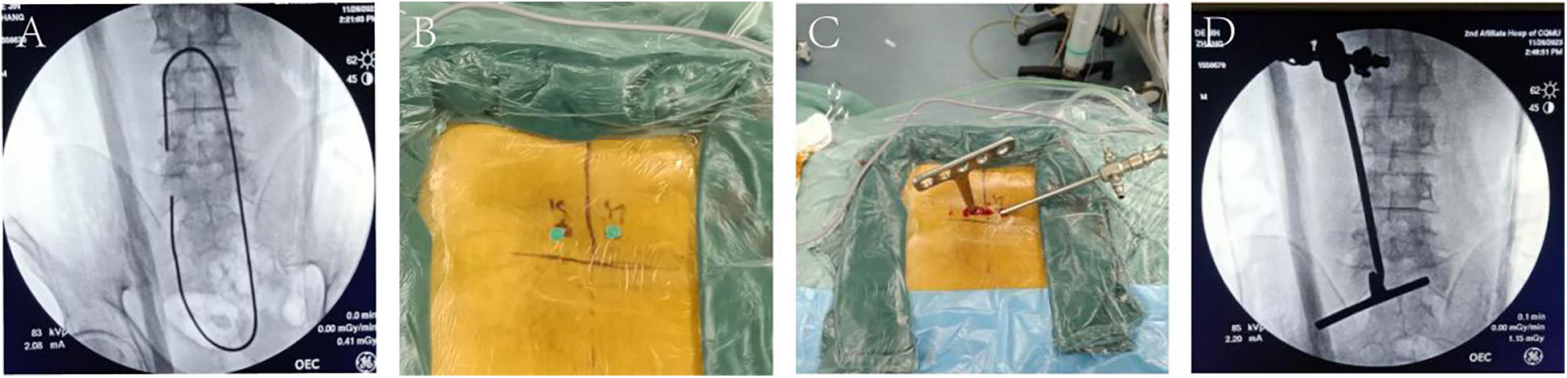
Localization of the UBE-LIF process. **(A,B)** Preoperative localization of the patients. **(C,D)** Surgical operation channel.

UBE-LIF group: After anesthesia and skin disinfection, the patient was placed in a prone position on the operating table. X-ray C-arm fluoroscopy was used to locate the upper pedicle and lower pedicle before the operation (Figure 2A). The projection points of the left vertebral arch were punctured with a special puncture needle (Figure 2B). Two longitudinal incisions of approximately 2.5 and 0.5 cm were made for the endoscopic channel and the surgical channel, respectively (Figure 2C).

**Figure 3:**
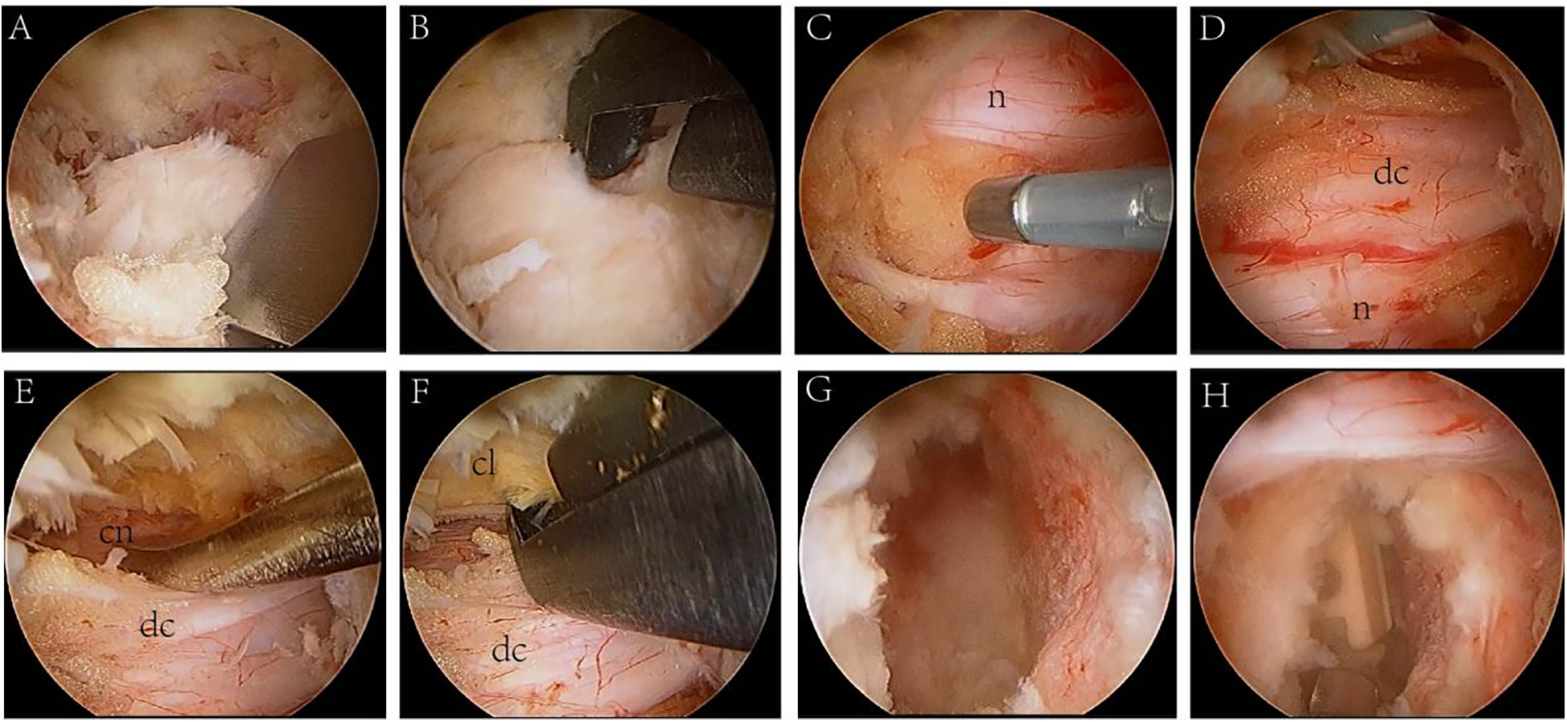
Endoscopic surgery view. **(A,B)** The inferior articular process and part of the superior articular process were removed using an osteotome and a lamina rongeur. **(C,D)** Exposed nerve root (n) and dural sac (dc). **(E)** The dural sac was protected, and the contralateral nerve root (cn) was exposed. **(F)** The hypertrophic contralateral ligamentum flavum (cl) was removed. **(G)** Nucleus pulposus forceps was used to scrape the intervertebral space. **(H)** Implant cage.

**Figure 4:**
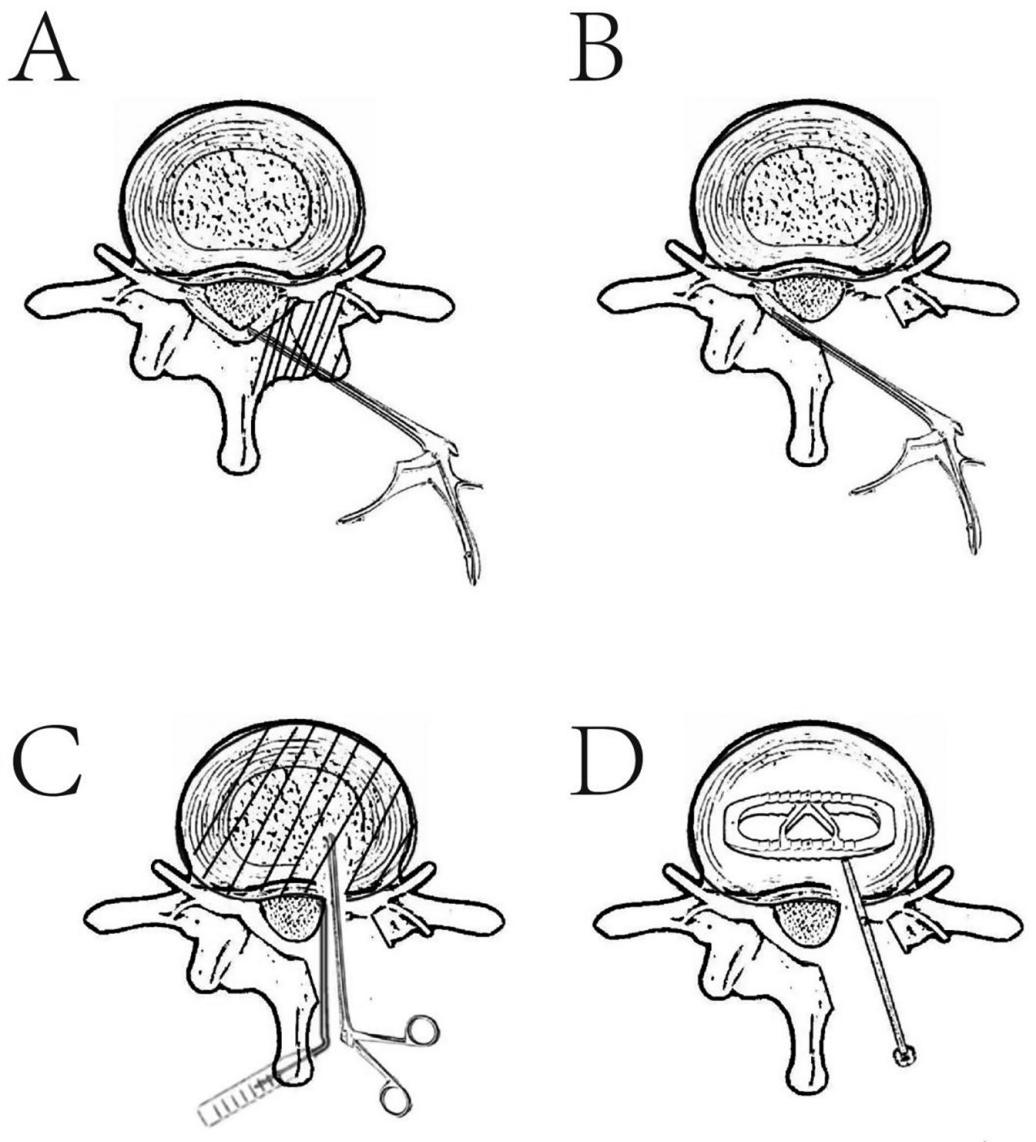
Simple schematic diagram of unilateral biportal endoscopic lumbar interbody fusion (UBE-LIF) surgery. **(A)** The affected vertebral plate, superior articular process, and ipsilateral ligamentum flavum were removed using an osteotome, and the ipsilateral recess was decompressed. **(B)** The ipsilateral and contralateral hypertrophic ligamentum flavum were removed , and the bilateral lateral recess and spinal canal were decompressed. **(C)** The dural sac was pulled to one side using the nerve root retractor and the nucleus pulposus was removed using the nucleus pulposus forceps. **(D)** Cage implantation in the intervertebral disc.

The interlaminar space and ligamentum flavum were fully exposed. The lower articular process of the left upper vertebra and the upper articular process of the lower tail vertebra were removed using an osteotome and a lamina rongeur. The hypertrophic ligamentum flavum was removed, and part of the lamina was removed with a grinding drill to expand the spinal canal. The intervertebral disc was fully exposed, the annulus fibrosus was cut off with a reamer, and the intervertebral disc and cartilage endplate were scraped with a reamer and a scraper. Continue to remove part of the lamina, part of the articular facet and ligamentum flavum to the contralateral side to decompress the contralateral nerve root and dural mater. The fusion cage (Double Medical Technology Inc.; bullet-shaped, polyether ether ketone material), filled with autologous bone particles, was implanted through the bone graft channel. Subsequently, C-arm fluoroscopy was used to locate the bilateral pedicles of L5 and S1. The pedicle was punctured with a special puncture needle, and the skin was cut approximately 1.5 cm. When the anteroposterior fluoroscopy tip was located at the inner edge of the pedicle and the lateral fluoroscopy did not exceed the posterior edge of the vertebral body, the guide wire and the expansion channel were placed, and then the pedicle screw was percutaneously placed. The position of the screw was available during the operation. The bilateral upper connecting rod and the top wire were locked and fixed, and the intervertebral space was pressurized and fixed. The wound was rinsed with a large amount of normal saline and sutured, and then a drainage tube was placed. All patients felt good in both lower limbs after the operation and returned to the ward (Figures 2–4).

PLIF group: After general anesthesia and disinfection, the position commonly used in spinal surgery was adopted. A posterior median incision of the waist was performed with the surgical segment as the center. The bilateral lamina and facet joints were exposed. The lamina of the upper vertebral body and the medial part of the bilateral articular process was removed. The lateral recess was decompressed on both sides, and the nerve root was released. The dural sac and nerve root were exposed by removing the ligamentum flavum. The intervertebral disc was identified using a curette and nucleus pulposus forceps. A cage filled with autologous bone was implanted. C-arm fluoroscopy was used to show that the cage was in good position. Pedicle screw (Double Medical Technology Inc.) fixation was used.

### Postoperative treatment

Both groups were given a postoperative course of antibiotics to prevent infection, alongside the appropriate use of mannitol and non-steroidal anti-inflammatory drugs to relieve pain and detumescence. Blood analysis and biochemical examination were performed on the first day after the respective operations. Every 3 days until discharge, blood counts, liver function, kidney function, and C-reactive protein were rechecked. The drainage volume was recorded per day, and the drainage tube was removed when the drainage volume was less than 50 ml/24 h.

### Outcome measures

Data on the operation time, postoperative drainage volume (POV), hemoglobin loss, hospital stay, and complication rate were compared between the two groups. The MacNab criteria, visual analog scale (VAS) score, and Oswestry Disability Index (ODI) score were compared between the two groups before surgery; 1 week, 3 months, and 6 months after surgery; and at the last follow-up.

### Statistical analysis

The data analysis was performed using SPSS version 27.0 software (SPSS, Inc., Chicago, IL, USA). The continuous data were compared using the independent-sample *t*-test. The chi-square test was used to compare categorical data and Fisher’s test was used when the amount of data in the two groups was less than five. Statistical significance was defined as *P* < 0.05.

## Results

### Demographics characteristics

In total, 50 patients were eligible for the study. The mean age of the 21 patients (12 men and 9 women) who underwent the UBE-LIF procedure was 67.14 ± 10.27 years (ranging from 49 to 81 years). The mean age of the 29 patients (17 men and 12 women) who underwent the PLIF procedure was 69.55 ± 9.66 years (ranging from 43 to 82 years). There was no significant difference between the two groups in age, gender, body mass index, level of disc herniation, hypertension, and diabetes (*P* > 0.05) (Table 1). The two groups of patients had single-segment lumbar spinal stenosis.

**Table 1:** Demographics and baseline characteristics of the two groups.

Variable	UBE-LIF (*n* = 21)	PLIF (*n* = 29)	*P*
Age (years)	67.14 ± 10.27	69.55 ± 9.66	0.401
Gender			0.917
Male	12	17	
Female	9	12	
BMI (kg/m^2^)	24.15 ± 2.84	24.19 ± 2.69	0.951
Lesion level			0.433
L3/4	1	4	
L4/5	15	21	
L5/S1	5	4	
Hypertension			0.340
Yes	8	15	
No	13	14	
Diabetes			0.485
Yes	4	8	
No	17	21	
Follow-up duration (months)	12.86 ± 0.85	12.48 ± 0.63	0.081

BMI, body mass index.

### Operative data

The hemoglobin loss, drainage volume on the first day after operation, and hospitalization time in the UBE-LIF group were lower than the PLIF group (*P* < 0.05). The operation time in the UBE-LIF group was significantly longer than that in the PLIF group (*P* < 0.05) (Table 2).

**Table 2:** Comparison of the surgical details between the UBE-LIF and PLIF groups.

Variable	UBE-LIF (*n* = 21)	PLIF (*n* = 29)	*P*
Operation time (min)	199.81 ± 24.67	162.72 ± 14.38	<0.001
Hemoglobin loss (g/L)	13.76 ± 4.61	21.14 ± 7.31	<0.001
POV (ml)	75.71 ± 66.97	175.48 ± 58.81	<0.001
Hospital stay (days)	9.81 ± 1.83	12.31 ± 1.71	<0.001

POV, postoperative drainage volume.

Only the drainage volume of the first day after operation was included.

### Clinical outcomes

**Figure 5:**
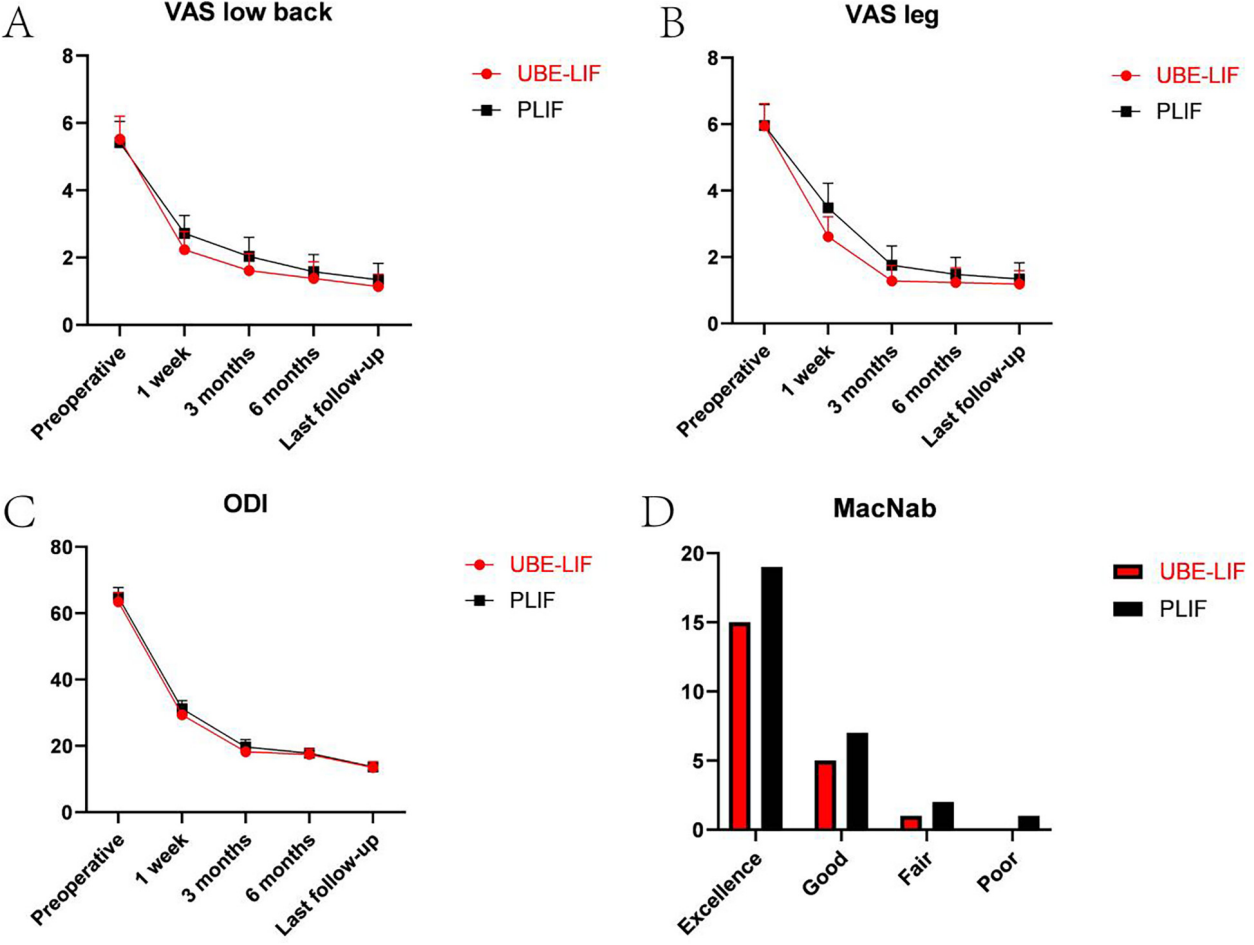
Preoperative and postoperative (1 week, 3 months, 6 months, and at the last follow-up) VAS low back, VAS leg, ODI, and MacNab. **(A)** Preoperative and postoperative (1 week, 3 months, 6 months, and at the last follow-up) VAS low back. **(B)** Preoperative and postoperative (1, 3, 6, and 12 months) VAS leg. **(C)** Preoperative and postoperative (1, 3, 6, and 12 months) ODI. **(D)** Postoperative MacNab.

There was no significant difference in VAS score for leg pain or lower back pain and ODI score between the two groups before the respective operations (*P* > 0.05). After evaluating the 1-week and 3-month postoperative results, the VAS score and ODI score in the UBE-LIF group were significantly lower than those in the PLIF group. At 6 months and the last follow-up, there were no significant differences in VAS score or ODI score between the UBE-LIF group and the PLIF group. There was no significant difference in the MacNab score between the two groups (*P* > 0.05). Both the UBE-LIF group and the PLIF group had significant clinical efficacy (Tables 3, 4, Figure 5).

**Table 3:** Comparison of clinical effectiveness between the UBE-LIF and PLIF groups.

Variable	UBE-LIF (*n* = 21)	PLIF (*n* = 29)	*P*
VAS leg			
Preoperative	5.95 ± 0.67	5.97 ± 0.63	0.944
1 week	2.62 ± 0.59^a^	3.48 ± 0.74^a^	<0.001
3 months	1.29 ± 0.46	1.76 ± 0.58	0.002
6 months	1.24 ± 0.44	1.48 ± 0.51	0.081
Last follow-up	1.19 ± 0.40	1.34 ± 0.48	0.239
VAS lower back			
Preoperative	5.52 ± 0.68	5.41 ± 0.63	0.557
1 week	2.24 ± 0.54^a^	2.72 ± 0.53^a^	0.003
3 months	1.62 ± 0.49	2.03 ± 0.57	0.010
6 months	1.38 ± 0.49	1.59 ± 0.50	0.158
Last follow-up	1.14 ± 0.36	1.34 ± 0.48	0.112
ODI score			
Preoperative	63.43 ± 2.84	64.93 ± 2.85	0.072
1 week	29.33 ± 1.56^a^	31.21 ± 2.43^a^	0.002
3 months	18.24 ± 1.33	19.72 ± 2.17	0.004
6 months	17.43 ± 1.63	17.83 ± 1.44	0.365
Last follow-up	13.52 ± 1.69	13.72 ± 1.25	0.632
MacNab			0.473
Excellence	15	19	
Good	5	7	
Fair	1	2	
Poor	0	1	
Excellence/good rate (%)	95.24	89.66	

^a^ indicates that there was a statistically significant difference between the score 1 week after surgery and the preoperative score.

**Table 4:** Comparison of the postoperative complication and failed intervertebral fusion rates between the UBE-LIF and PLIF groups.

Variable	UBE-LIF (*n* = 21)	PLIF (*n* = 29)	*P*
Failed infusion 6 months after surgery	2	3	0.924
Failed infusion 12 months after surgery	1	2	0.754
Complications, *n* (%)	0 (–)	2 (6.8%)	0.219

### Postoperative complications

The summary of complications in each group is presented in Table 4. No loosening, fracture or subsidence of screws occurred in the two groups after the respective operations. Among the patients who underwent PLIF, one case had poor wound healing (3.4%) and one case had poor pain relief in the lower limbs (3.4%). There were no significant differences in postoperative fusion failure and postoperative complications between the two groups (*P* > 0.05).

### Typical cases

**Figure 6:**
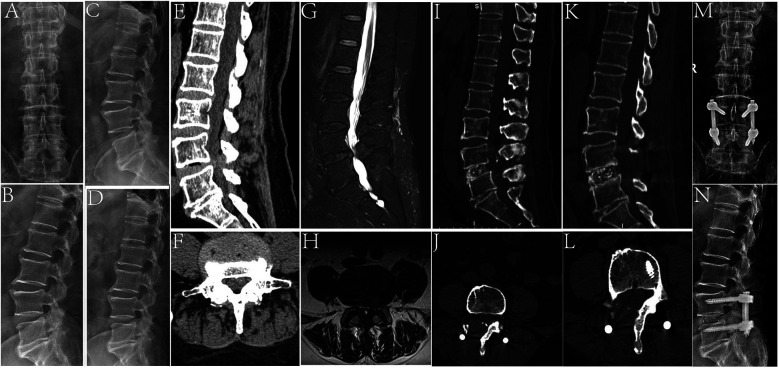
A 76-year-old male patient who underwent UBE-LIF. **(A,B)** Preoperative anteroposterior and lateral x-ray images. **(C,D)** Preoperative lumbar imaging in the dynamic position. **(E,F)** Preoperative lumbar MRI T2 images in the sagittal and transverse planes showed L4/5 spinal stenosis. **(G,H)** Preoperative lumbar CT in the sagittal and transverse positions. **(I,J)** Postoperative lumbar CT in the sagittal and transverse positions showed complete decompression of the spinal canal. **(K,L)** The last follow-up of lumbar CT in the sagittal and transverse positions showed no significant abnormality in the surgical area and the vertebral body had fused. **(M,N)** Postoperative x-ray showed that the screw was fixed in place.

A 76-year-old male patient who had repeated lower back pain with lower limb pain for half a year underwent UBE-LIF surgery. The preoperative imaging examination revealed L4/5 spinal stenosis. After L4–L5 UBE-LIF surgery, the patient was discharged after the symptoms of lower back pain and claudication were relieved (Figure 6).

**Figure 7:**
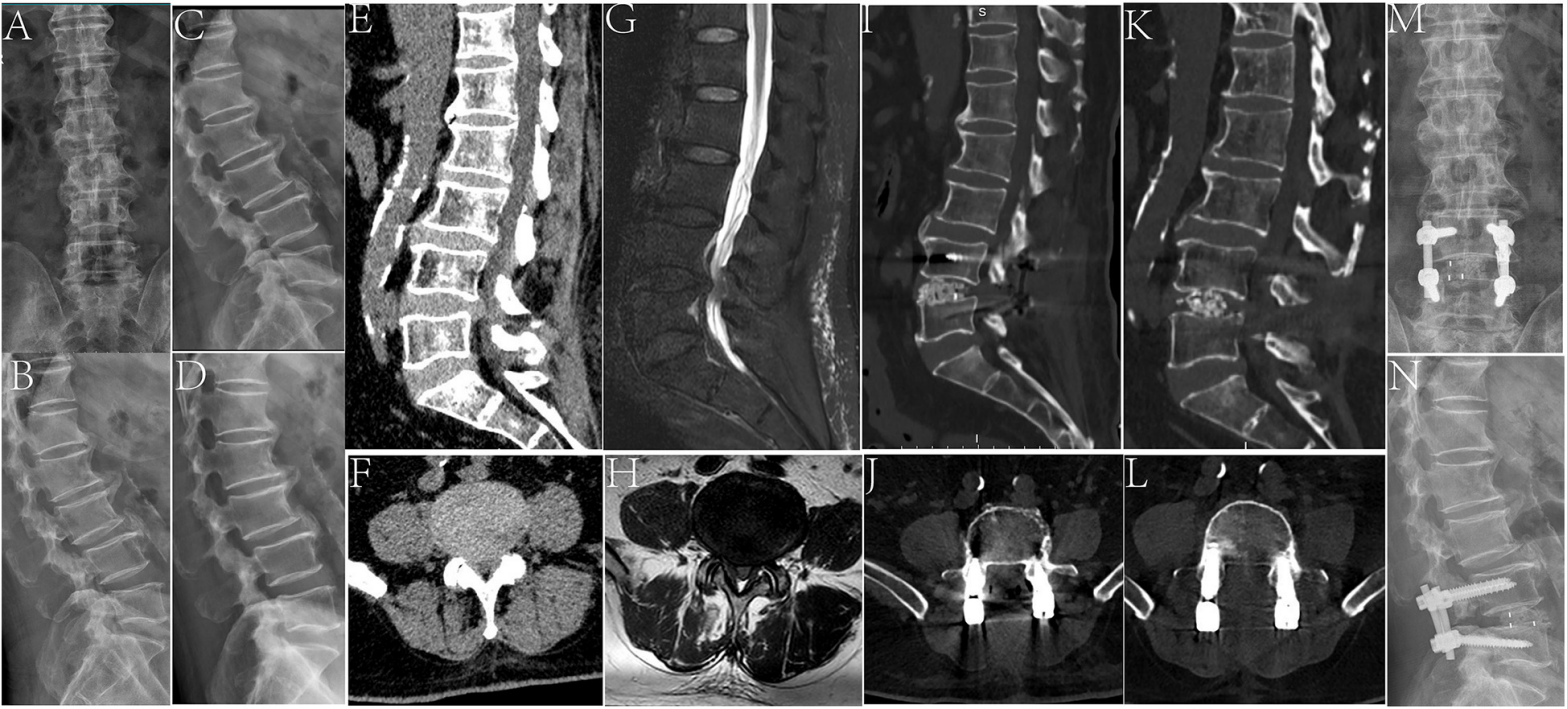
An 80-year-old male patient who underwent PLIF surgery. **(A,B)** Preoperative lumbar lateral x-ray. **(C,D)** Preoperative lumbar imaging in the dynamic position. **(E,F)** Preoperative lumbar MRI T2 images in the sagittal and transverse planes showed L4/5 spinal stenosis. **(G,H)** Preoperative lumbar CT in the sagittal and transverse positions. **(I,J)** Postoperative lumbar CT in the sagittal and transverse positions showed that the spinal canal was completely decompressed and the screw was fixed in position. **(K,L)** The last follow-up lumbar CT images in the sagittal and transverse positions. **(M,N)** Anteroposterior and lateral x-ray images at the last follow-up.

An 80-year-old male patient with lower back pain for 5 years and aggravated lower back pain for 1 month was admitted to the hospital. The patient had suffered from fatigue of both lower limbs and intermittent claudication 1 month before admission. Imaging examination showed L4/5 spinal stenosis. After L4–L5 PLIF surgery, the patient was discharged after the symptoms of lower back pain and claudication were relieved (Figure 7).

## Discussion

In this study, all the patients in the PLIF group and the UBE-LIF group completed the respective operations. The UBE-LIF group was superior to the PLIF group in terms of hospital stay, intraoperative bleeding, hemoglobin loss, and postoperative drainage (Table 2). There was no significant difference in operation time between the two groups. The early VAS score and ODI score in the UBE-LIF group were lower than those in the PLIF group. The pain scores of the two groups were significantly reduced after the respective operations (Table 3). There was no significant difference in postoperative complications between the two groups.

LSS is caused by degeneration of the intervertebral disc, facet joint, and ligamentum flavum. LSS causes lower back pain, leg pain, leg numbness, and intermittent claudication, which harms the daily life and quality of life of the elderly (13). When conservative treatment fails, surgery is usually required to relieve symptoms. The traditional surgical treatment of LSS is open decompression and fusion (PLIF). Most patients achieve satisfactory results due to appropriate decompression. However, owing to injury to soft tissue, fusion surgery is associated with a large number of complications, such as bleeding, soft tissue injury, and adjacent segment degeneration (14). Biomechanical studies have demonstrated the importance of the posterior column, including the interspinous ligaments, facet joints, and capsules, in maintaining spinal stability. Therefore, minimizing damage to paraspinal muscles and posterior stabilizing structures is critical for positive long-term outcomes (15).

In a meta-analysis (9) to compare UBE-LIF and conventional posterior procedures, the early postoperative back VAS score (*P* = 0.002) and the ODI at 1 month postoperatively (*P* = 0.018) in the UBE-LIF group were significantly lower than those in the PLIF group. Furthermore, the postoperative complication rate (*P* = 0.553) in the UBE-LIF group and the PLIF group was not significantly different. Another meta-analysis conducted by Qi et al. (11) also showed that UBE-LIF was superior to open surgery in terms of back VAS score in the early postoperative period (*P* = 0.00001), and there was no significant difference in postoperative complications (including incomplete decompression) (*P* = 0.64). In the current study, the patients in the UBE-LIF group reported less pain in the early postoperative period and there was less intraoperative bleeding in comparison with the PLIF group. These results indicate that UBE-LIF is a less invasive procedure than conventional PLIF. This study has shown that when UBE-LIF is an equally valid decompression and stabilization treatment as PLIF, the former results in better early pain relief and the patients have a better quality of life.

Lumbar interbody fusion using uniportal endoscopy is commonly limited by the large and rigid cage. Modified grafts, such as mesh filled with bone morphogenetic protein, are being considered by some researchers for utilization. However, the interbody fusion material may not provide sufficient stability without a rigid cage. The cage placement with endoscopy is effectively performed using the biportal endoscopic technique (16). A retractor designed to protect the thecal sac avoids the risk of nerve injury in the blind space during cage insertion from the skin to the endoscopic field. Many different sizes are possible as the cage can pass through the working channel. The ability to place larger fusers and adjust the fuser angle more easily may explain the higher fusion rate in UBE-LIF (17, 18).

The learning curve for endoscopic surgery reflects, to a certain extent, the difficulty of the surgery and the speed at which the skills can be mastered in a given period (18). A learning curve analysis study by Guo et al. (19) showed that mastery of ULIF surgery requires at least 29 operations and at least 41 operations to achieve a stable surgical success rate. Although the operative time cannot simply be used to define the learning curve, it can be seen that the operative time of the learning and mastery phases (29 cases) was significantly different (175.38 ± 34.23 min vs. 133.55 ± 22.76 min). Postoperative complications were defined as the occurrence of surgical failure. The learning curve based on the failure rate of surgery showed that the incidence of complications (2.6%) was significantly lower than that of the learning phase (17.07%) after 41 surgery cases (19). The potential disadvantages of UBE-LIF are a long operating time and a steep learning curve. UBE-LIF is based on UBE surgery. Performing UBE-LIF without UBE experience may have a negative effect on surgical outcomes. The experience of at least 90 UBLD cases must be accumulated prior to LIF surgery to facilitate adaptation to the UBE-LIF technique (20). Novice surgeons should select easier cases in the early stages to shorten the learning curve.

UBE-LIF is also inevitably associated with some bleeding, especially when the ligamentum flavum and upper joint are removed, which can lead to delayed recovery and associated complications. This study found that intraoperative bleeding and postoperative drainage were significantly lower in the UBE-LIF group than in the PLIF group. Although continuous fluid irrigation plays a crucial role in controlling epidural and bone surface bleeding, the pressure of the irrigation and continuous drainage should be monitored to prevent postoperative pain (9). The working area created between the endoscope and the tissue requires continuous irrigation to maintain a satisfactory surgical field of view. However, a large amount of irrigation fluid and irrigation pressure can compress the dural sac and lead to increased lateral cranial pressure, resulting in complications such as headache, seizures, and even death (21).

Compared to the indirect decompression effect of the lateral and anterior approaches, the combination of percutaneous UBE and minimally invasive transforaminal lumbar interbody fusion (TLIF), with maximum preservation of normal muscle and ligament structure, can achieve direct decompression through a unilateral approach for discectomy, facetectomy, and bilateral laminectomy (10). Although traditional open TLIF and PLIF are effective methods for treating degenerative lumbar spinal stenosis, they can cause damage to muscle and ligament structures, leading to back syndromes. MIS-TLIF, however, involves unilateral laminectomy, bilateral decompression, discectomy, and bone graft with cage placement. Percutaneous endoscopic posterior lumbar interbody fusion (PE-PLIF) is a single-entry endoscopic technique. The PE-PLIF procedure utilizes a working cannula that is positioned and angled similarly to the ULBD under endoscopy, allowing for the completion of ULBD during the PE-PLIF procedure. However, visualization of the opposite side can be challenging, particularly when preparing the endplate. Additionally, the procedure can be time-consuming. In contrast, the UBE-LIF technique allows for the retention of bilateral muscle connections as no tubular retractor is used. In UBE-LIF cases, it is easier to identify and prepare the final version, allowing for the elimination of complete cartilage (22, 23). Intervertebral fusion and bilateral decompression can be completed simultaneously without the need for additional contralateral surgery. Bilateral decompression through a unilateral approach can minimize the trauma to the structure of the paraspinal muscle ligament. It can fully decompress the lateral recess and intervertebral foramen and minimize nerve damage; the stability of the spine is maintained by minimizing the incision in the intervertebral foramen (24). During UBE-LIF surgery, bilateral decompression is performed to alleviate the patient's lower limb symptoms. This technique reduces damage to the patient's back muscles, bones, and ligaments compared to PLIF, while achieving similar long-term clinical results.

Lumbar fusion is an effective technique for the treatment of degenerative lumbar diseases (4). For patients with spinal stenosis caused by degenerative lumbar changes without spondylolisthesis, scoliosis, or nerve compression confined to the central or lateral recess of the spinal canal, simple decompression can usually achieve satisfactory clinical results (25). When there is lumbar instability or deformity (such as spondylolysis), spondylolisthesis, intervertebral foramen stenosis requiring extensive decompression, recurrence of symptoms after simple decompression, or accompanied by chronic lower back pain symptoms, fusion surgery is more likely to be selected to stabilize the diseased vertebral body (26, 27). Some studies suggest that for patients with simple stenosis without spinal spondylolisthesis, there is no significant difference in long-term clinical efficacy between fusion surgery and simple decompression surgery, but is more likely to get complicated. Patients with lumbar instability confirmed by preoperative dynamic lumbar positioning are candidates for spinal fusion surgery (28).

There are some common limitations in this study, such as short follow-up time and relatively small sample size. Radiological parameters such as intervertebral space height were not included in this paper.

## Conclusion

This study showed that PLIF and UBE-LIF are safe and effective in the treatment of LSS. UBE-LIF can also be used to perform bilateral decompression through a unilateral approach. In addition, compared with traditional PLIF, UBE-LIF has advantages in perioperative indicators and early postoperative pain scores, which may be related to less muscle damage during the surgery. However, due to the limitations of this study, studies with more long-term follow-up, more evaluation indicators, and a larger sample size are still needed to explore the difference in clinical efficacy between UBE-LIF and PLIF.

## Data Availability

The raw data supporting the conclusions of this article will be made available by the authors, without undue reservation.
